# Distributed representation and one-hot representation fusion with gated network for clinical semantic textual similarity

**DOI:** 10.1186/s12911-020-1045-z

**Published:** 2020-04-30

**Authors:** Ying Xiong, Shuai Chen, Haoming Qin, He Cao, Yedan Shen, Xiaolong Wang, Qingcai Chen, Jun Yan, Buzhou Tang

**Affiliations:** 1 Department of Computer Science, Harbin Institute of Technology, Shenzhen, Guangdong China; 2 Peng Cheng Laboratory, Shenzhen, Guangdong China; 3 Yidu Cloud (Beijing) Technology Co., Ltd, Beijing, China

**Keywords:** Clinical semantic textual similarity, Gated network, Distributed representation, One-hot representation

## Abstract

**Background:**

Semantic textual similarity (STS) is a fundamental natural language processing (NLP) task which can be widely used in many NLP applications such as Question Answer (QA), Information Retrieval (IR), etc. It is a typical regression problem, and almost all STS systems either use distributed representation or one-hot representation to model sentence pairs.

**Methods:**

In this paper, we proposed a novel framework based on a gated network to fuse distributed representation and one-hot representation of sentence pairs. Some current state-of-the-art distributed representation methods, including Convolutional Neural Network (CNN), Bi-directional Long Short Term Memory networks (Bi-LSTM) and Bidirectional Encoder Representations from Transformers (BERT), were used in our framework, and a system based on this framework was developed for a shared task regarding clinical STS organized by BioCreative/OHNLP in 2018.

**Results:**

Compared with the systems only using distributed representation or one-hot representation, our method achieved much higher Pearson correlation. Among all distributed representations, BERT performed best. The highest Person correlation of our system was 0.8541, higher than the best official one of the BioCreative/OHNLP clinical STS shared task in 2018 (0.8328) by 0.0213.

**Conclusions:**

Distributed representation and one-hot representation are complementary to each other and can be fused by gated network.

## Background

Electronic Health Records (EHRs) that record patients’ complete information, including family history, general situation, chief complaint, examination, lab test, diagnosis, assessment, and plan, etc., have been widely used to help medical experts to improve processes of care on patient outcomes. The key to secondary use of EHRs lies in high quality. However, the quality of EHRs has met challenges such as frequent use of copy-and-paste, templates, and smart phrases which lead to bloated or erroneous clinical notes [1]. A study of 23,630 clinical notes written by 460 clinicians showed that 46% of the text in the clinical records copied other clinical records, 36% was imported from templates, and only 18% was manually entered [2]. To aggregate data from diverse sources and minimize data redundancy, BioCreative/OHNLP organized a shared task to evaluate the semantic similarity between text snippets (also called sentences in this paper) of clinical texts in 2018. In this shared task, the similarity between two clinical text snippets ranged from 0 to 5, where 0 means that the two clinical text snippets are not semantically similar at all, and 5 indicates that the two clinical text snippets are entirely equal. In the past few years, SemEval workshop has launched STS shared task in the general domain many times [3–8]. In the clinical area, BioCreative/OHNLP first organized an STS shared task in 2018.

As many NLP applications such as QA, IR, etc. usually used STS as a core component, large quantities of researchers have contributed to STS and achieved great success. STS is a typical regression problem, and how to model sentence pairs is the key to solutions. There are two main types of representations to model sentence pairs: one-hot representation and distributed representation. The one-hot representation that depends on manually-crafted features suffers from sparsity. The distributed representation that learns dense real-value vector from unlabeled data automatically by neural networks have shown great potentialities. Most studies focus on one type of representations. In this paper, we proposed a novel framework to fuse the two types of representations using a gated network. In the case of distribution representations, we compared some current state-of-the-art neural networks such as CNN, Bi-LSTM, and BERT. To evaluate our method, we conducted experiments on the clinical STS corpus of BioCreative/OHNLP 2018 by comparing our method with the methods that only using one type of representation and the official best method on the clinical STS shared task. Experimental results showed that: 1) the proposed method achieved much higher Pearson correlation than the methods only using one type of representations. 2) BERT performed better than other distributed representations. 3) Our method achieved the highest Pearson correlation of 0.8541, higher than the best official one of the clinical STS shared task (0.8328) by 0.0213.

## Related work

There are two main types of sentence representation: (1) sparse one-hot representation based on manually extracted features. (2) densely distributed representation learnt from large labeled data. Within a long period, there have been a large number of feature extraction methods proposed to represent sentence by one-hot vector. Gomaa et al. [9] summarized several types of features and various similarity computation methods: string-based similarity computation methods such as N-gram [10–12], corpus-based similarity methods [13–16] and knowledge-based similarity computation methods [17–19]. In recent years, neural networks have became mainstream methods for sentence representation and STS. Bromley et al. [20] firstly presented a Siamese architecture to encode sentence pairs. Based on previous work, Mueller et al. [21] used Siamese recurrent architecture learning sentence representation. Tang et al. [22] used deep belief network to learn sentence representation. He et al. [23] proposed a novel pairwise word interaction method to measure the sentence semantic similarity. Gong et al. [24] further hierarchically extracted semantic features from interaction space. Tai et al. [25] used tree-structured LSTM to improve the sentence representation. Subramanian et al. [26] used transfer learning to learn sentence representation. In recent years, neural language models have been also ultilized for sentence representation, such as ELMo [27] and GPT [28]. Some researchers extracted features at different granularities and combined them with distributed representations, such as He et al. [29] and Wang et al. [30]. Ji et al. [31] combined the features with distributed representation, our work was similar to Ji’s work, but we used a novel gate to choose how to combine one-hot representation and distributed representation.

## Methods

### Task definition

Formally, the clinical STS task is to determine the similarity of a pair of given sentences, denoted by *sim*(*s*_1_, *s*_2_), where *s*_1_ is a sentence of length *m* and *s*_2_ is a sentence of length *n*. We used *s*_*ij*_ to denote the *j*-th word of *s*_*i*_. In this study, the similarity of a sentence pair ranged from 0 to 5, where 0 represents the two sentences are not semantically similar, and 5 represents the two sentences are semantically equal. Besides, we used *D* and *O* to describe a sentence’s distributed representation and one-hot representation respectively.

### Dataset

**Fig. 1:**
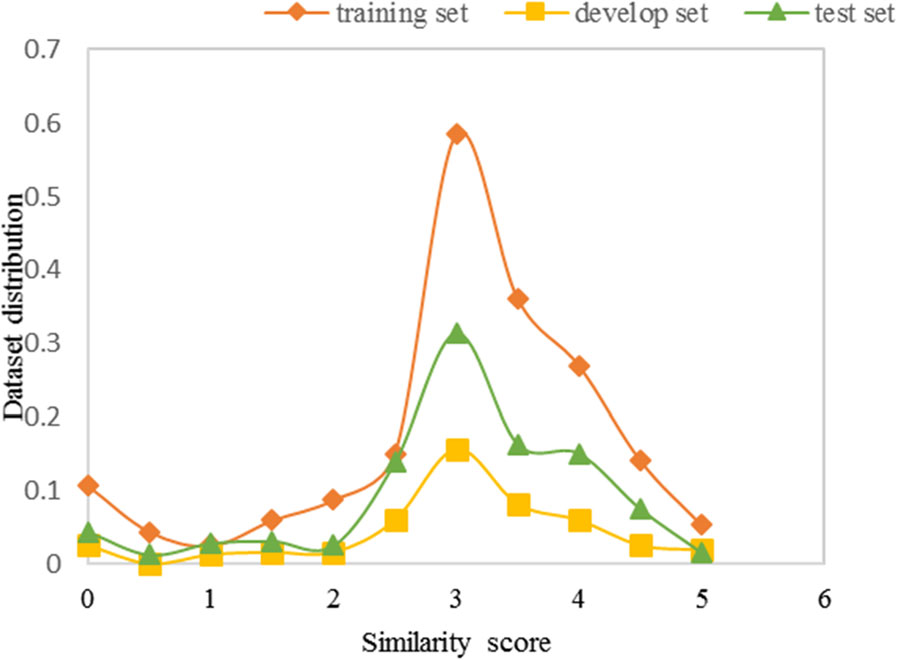
Fractional similarity interval distribution in the training, develop and test sets

The BioCreative/OHNLP organizer manually annotated 750 sentence pairs with semantic similarity ranging from 0 to 5 for system development and 318 sentence pairs for system test. We further divided the 750 sentence pairs into a training set and a develop set using stratified sampling to guarantee that the develop set is a representative of the overall dataset. Figure 1 shows the fractional similarity interval distribution in the training, development and test sets, and Table 1 lists some annotated examples.

**Table 1:** Annotated examples

Score	Example
0	*s*_1_: discus necessity member healthcare team male female participate procedure *s*_2_: report represent interpretation original data trace store electronic record esophageal laboratory
1	*s*_1_: mother blood type o + hepatitis b negative hiv negative found gb positive *s*_2_: patient undergone genetic test found brca1 2 negative well bart negative
2	*s*_1_: patient discharge home ambulate without assistance discharge instruction give patient *s*_2_: patient left without see ambulate without assistance family drive accompany husband wife
3	*s*_1_: negative cardiovascular review system historian denies chest pain dyspnea exertion *s*_2_: negative cardiovascular review system historian denies chest pain diaphoresis syncope palpitation
4	*s*_1_: patient education ready learn apparent learn barrier identify learn preference include listen *s*_2_: assistance somali interpreter ready learn apparent learn barrier identify learn preference include listen
5	*s*_1_: nurse visit ten minute half spent counsel point test *s*_2_: nurse visit ten minute half spent consultation point test

### Data processing

We preprocessed each sentence as follows: 1) used NLTK tool (http://www.nltk.org/) for tokenization and lemmatization; 2) converted Arabic numerals into English numbers. For example, the sentence “Indication, Site, and Additional Prescription Instructions: Apply 1 patch every 24 hours; leave on for up to 12 hours within a 24 hour period” became “indication site additional prescription instruction apply one patch every twenty four hour leave twelve hour within twenty four hour period” after preprocessing.

### Distributed representation and one-hot representation fusion

**Fig. 2:**
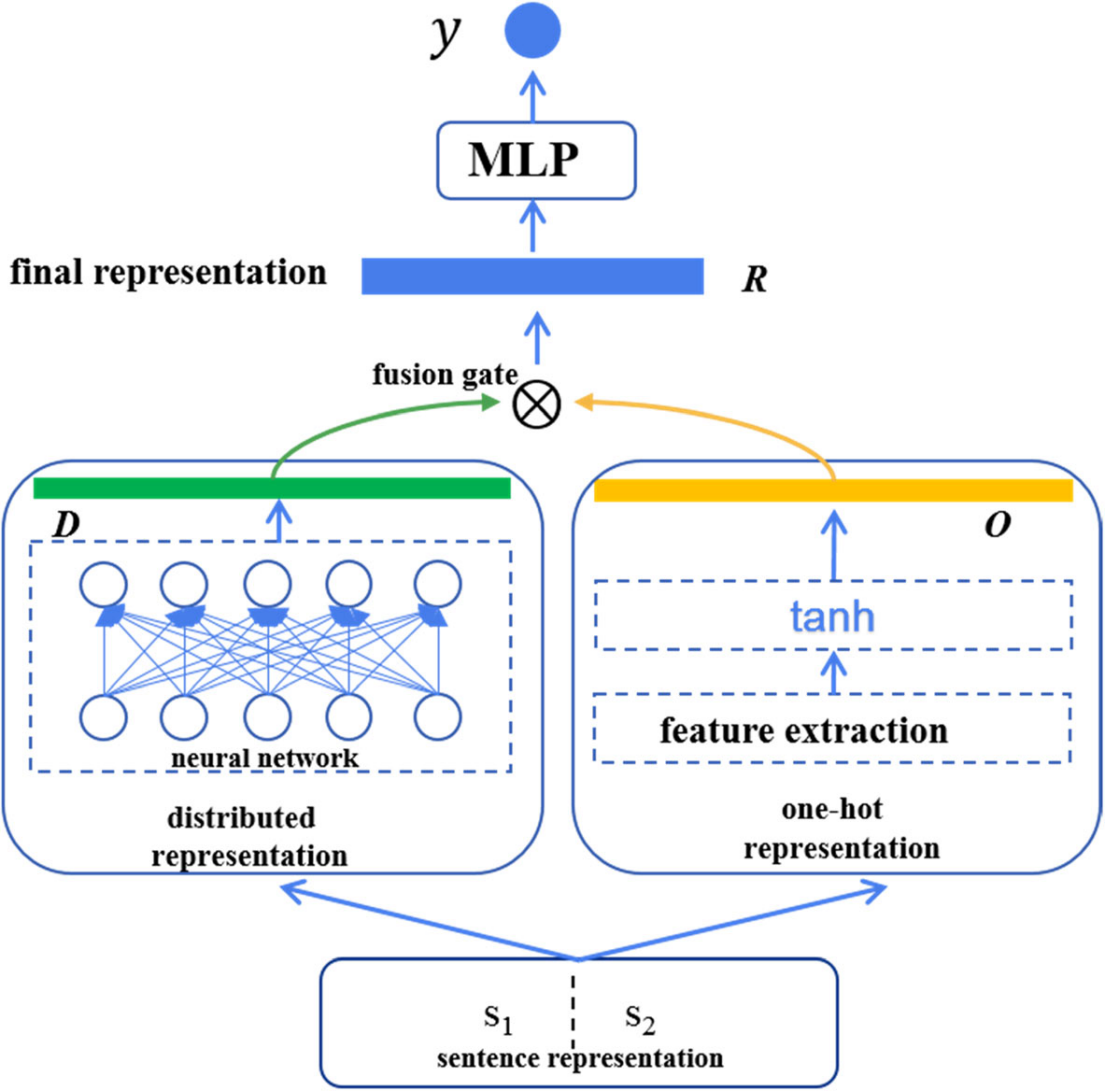
Overview architecture of our distributed representation and one-hot representation fusion system based on gated network

Figure 2 shows an overview architecture of our distributed representation and one-hot representation fusion system based on a gated network for the clinical STS task of BioCreative/OHNLP 2018 (i.e., task2). The system consists of three components: (1) sentence pair representation – distributed representation and one-hot representation; (2) representation fusion with gated network; (3) neural network to compute sentence similarity. We described some of them in the following sections in detail.

### Distributed representation

In this study, we investigated three types of distributed representations: Siamese CNN [32], Siamese RNN [21] and BERT [33], where Siamese CNN and Siamese RNN are two popular neural networks used to represent sentence pair, while BERT is a new language representation method proposed recently.

1. **Siamese CNN** is composed of two CNNs, each of which represents a sentence, and the two CNNs share weights. The representation of sentence pair (*s*_1_, *s*_2_) is obtained as follows:

1 $$ {\displaystyle \begin{array}{c} CNN\left(\bullet \right)=a\mathrm{vg}\_ pool\left( convolution\left(\bullet \right)\right)\\ {}{D}_{cnn}=\left[ CNN\left({s}_1\right), CNN\left({s}_2\right)\right],\end{array}} $$

where *a*vg _ *pool* is the average pooling operation, *convolution* is the convolution operation, *s*_1_ and *s*_2_ are the two input sentences.

(2) **Siamese RNN**, similar to Siamese CNN, is composed of two RNNs that represent each one sentence respectively and share weights. In our study, we adopted Bi-directional Long Short Term Memory (Bi-LSTM) networks as an implementation of RNN, where each word *i* at *s*_1_ and *s*_2_ is represented as:
  2 $$ \kern1em {\displaystyle \begin{array}{c}{\overrightarrow{h}}_i^{s_1}=\overrightarrow{LSTM}\left({\overrightarrow{h}}_{i-1}^{s_1},{s}_{1i}\right)\kern2.5em i=1,\dots, m\\ {}{\overleftarrow{h}}_i^{s_1}=\overleftarrow{LSTM}\left({\overleftarrow{h}}_{i+1}^{s_1},{s}_{1i}\right)\kern2.5em i=m,\dots, 1,\end{array}} $$
  3 $$ \kern3.75em {\displaystyle \begin{array}{c}\ {\overrightarrow{h}}_i^{s_2}=\overrightarrow{LSTM}\left({\overrightarrow{h}}_{i-1}^{s_2},{s}_{2i}\right)\kern2.5em i=1,\dots, n\\ {}{\overleftarrow{\ h}}_i^{s_2}=\overleftarrow{LSTM}\left({\overleftarrow{h}}_{i+1}^{s_2},{s}_{2i}\right)\kern2.5em i=n,\dots, 1,\end{array}} $$

where $\overrightarrow{LSTM}$ and $\overrightarrow{LSTM}$ are the forward and backward LSTMs.

The sentence pair (*s*_1_, *s*_2_) is described as:

4 $$ {D}_{lstm}=\left[{\overrightarrow{h}}_m^{s_1},\kern0.5em {\overleftarrow{h}}_1^{s_1},\kern0.5em {\overrightarrow{h}}_n^{s_2},\kern0.5em {\overleftarrow{\ h}}_1^{s_2}\right] $$

(3) **BERT (B**idirectional **E**ncoder **R**epresentations from **T**ransformers**)** is a language representation method to obtain deep bidirectional representations of sentences by jointly conditioning on both left and right context in all layers from free text unsupervised. In our study, the representation of a sentence pair (*s*_1_, *s*_2_) was denoted by

5 $$ {D}_{bert}= BERT\left(\left[{s}_1,{s}_2\right]\right) $$

We trained a new BERT model on MIMIC III starting from the pre-trained model released by Google (https://github.com/google-research/bert).

### One-hot representation

We followed Tian’s work [34] to extract the following two types of features: (1) Sentence-level features: IDF (inverse document frequency) [35] and sentence length; (2) Sentence pair-level features: *N*-gram overlaps defined in eq. (6), and distances or similarities between the two input sentences calculated by cosine, Manhattan, Euclidean, Chebyshev, polynomial kernel, RBF kernel, Laplacian kernel and sigmoid kernel after each sentence is represented by the average vector of all words’ embeddings (https://github.com/mmihaltz/word2vec-GoogleNews-vectors).

6 $$ NGO\left({s}_1,{s}_2\right)=2\left(\frac{\left| Ngram\left({s}_1\right)\cap Ngram\left({s}_2\right)\right|}{\mid Ngram\left({s}_1\right)\mid +\mid Ngram\left({s}_2\right)\mid}\right) $$

where *Ngram*(*s*_*i*_) (*i* = 1,2) is a *N*-gram set extracted from *s*_*i*_. In our study, unigrams, bigrams and trigrams were considered.

### Fusion gate

Inspired by the gated network mechanism in variants of RNN such as LSTM and GRU (Gated Recurrent Unit), we introduced a gate to leverage distributed representation and one-hot representation. Before fusion, we adopted the tanh function as an activation function to convert the two types of representation into the same space. So that the final representation of sentence pair (*s*_1_, *s*_2_) *R* can be obtained in the following way:

7 $$ {D}_{norm}=\mathit{\tanh}\left({W}_d\bullet D+{b}_d\right) $$

8 $$ {O}_{norm}=\mathit{\tanh}\left({W}_o\bullet O+{b}_o\right) $$

9 $$ f=\sigma \left({W}_f\bullet \left[{D}_{norm},{O}_{norm}\right]+{b}_f\right) $$

10 $$ R=f\ast {D}_{norm}+\left(1-f\right)\ast {O}_{norm} $$

Where *W*_*d*_, *W*_*o*_, *W*_*f*_ are weights matrices; *b*_*d*_, *b*_*o*_, *b*_*f*_ are bias vectors; *σ* is the sigmoid activation function; *f* is leverage coefficient between the distributed representation and the one-hot representation.

### Experiments

We started from the baseline systems that only used one type of representations (distributed representation or one-hot representation), then concatenated the two types of representations, and finally fused the two types of representations with a gated network. All systems were evaluated on the clinical STS corpus of the BioCreative/OHNLP challenge in 2018, and Pearson correlation was used to measure the performance of the systems.

## Results

As shown in Table 2, the baseline system only using one-hot representation achieved much higher Pearson correlation than the baseline system just applying CNN or LSTM, but lower Pearson correlation than the baseline system only using BERT. The highest Pearson correlation of the baseline systems was 0.8461. When concatenating each distributed representation with the one-hot representation, we received higher Pearson correlation, indicating that the two types of representations are mutually complementary. For example, when we concatenated BERT with one-hot representation, we obtained a Pearson correlation of 0.8525, higher than the baseline system only using BERT by 0.0064 and the baseline system solely using one-hot representation by 0.0586. Instead of concatenating any distributed representation with one-hot representation, fusing them brought more significant improvement in Pearson correlation. The Pearson correlation difference between the systems that using concatenation strategy and fusion strategy ranged from 0.0016 to 0.0359. Among three distributed representations, our system achieved highest Pearson correlation of 0.8541 when using BERT for fusion, higher than the best official one of the BioCreative/OHNLP clinical STS shared task (0.8328) by 0.0213.

**Table 2:** Performance of systems on the clinical STS corpus of the BioCreative/OHNLP shared task in 2018

Method	Score Interval					
	[0,1]	[1, 2]	[2, 3]	[3, 4]	[4, 5]	Overall
Baseline						
One-hot	0.5567	0.2311	0.0998	0.2409	0.1167	0.7939
CNN	0.3960	−0.0850	−0.0090	0.0370	−0.0654	0.4444
LSTM	0.3920	−0.2945	0.2088	−0.0538	−0.0303	0.4275
BERT	0.7613	0.1206	0.2635	0.2530	0.1210	0.8461
Concatenation						
CNN + one-hot	0.5406	0.6917	0.1352	0.2539	0.0744	0.8083
LSTM+one-hot	0.5850	0.3415	0.2269	0.2173	0.2155	0.8030
BERT+one-hot	0.6684	0.3038	0.2309	0.2425	0.2203	0.8525
Fusion (gated network)						
CNN + one-hot	0.6973	0.2324	0.1675	0.2336	0.0864	0.8442
LSTM+one-hot	0.6253	0.3583	0.1869	0.2550	0.1018	0.8379
BERT+one-hot	0.6872	0.1605	0.3238	0.2822	0.1666	**0.8541**

## Discussion

In this study, we investigated three state-of-the-art distributed representation methods, that is, CNN, Bi-LSTM, and BERT, and proposed a novel framework based on a gated network to fuse distributed representation and one-hot representation of sentence pairs. Among the systems only using any one distributed representation or one-hot representation, the system using BERT achieved highest Pearson correlation, but the system using one-hot representation produced much higher Pearson correlation than the method using CNN or Bi-LSTM. Both concatenation and fusion of distributed representation and one-hot representation brought improvement, and the fusion with gated network performed better.

**Fig. 3:**
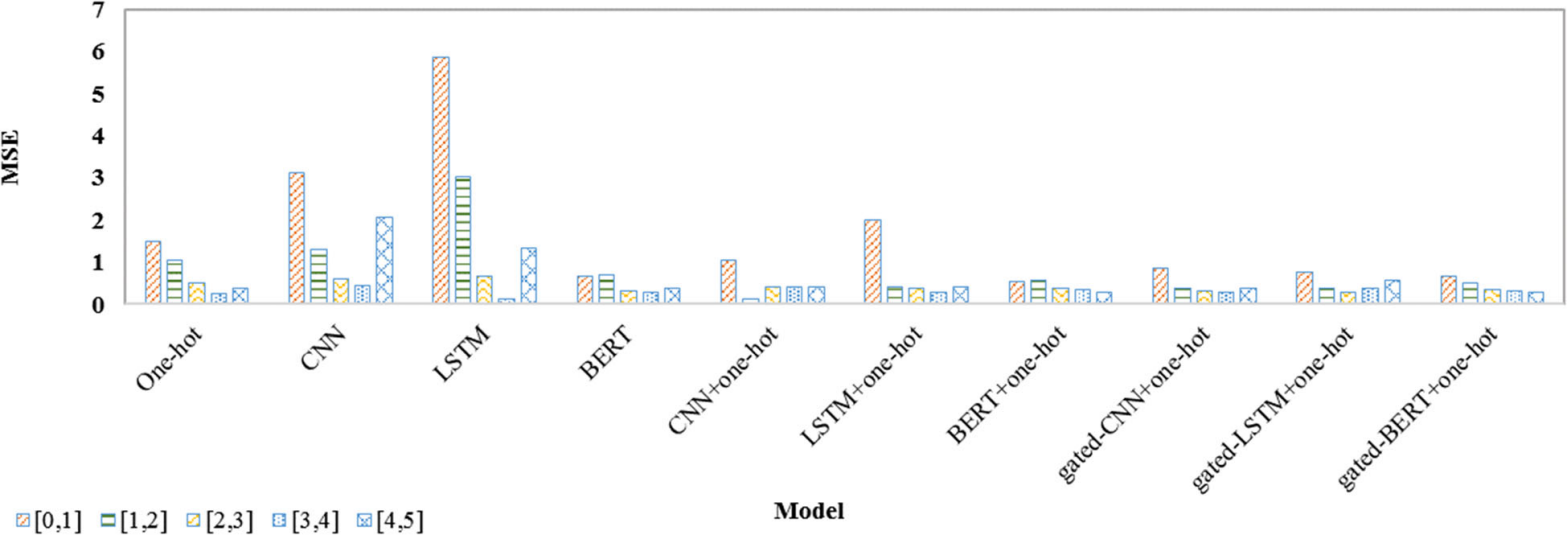
Mean Square Error (MSE) on fractional similarity intervals

The reason why the system using CNN or Bi-LSTM performed much worse than that using BERT or one-hot representation lies in the following two aspects: 1) the word embeddings used in CNN or Bi-LSTM were trained on a much smaller corpus than BERT; 2) one-hot representation had an advantage over CNN and Bi-LSTM on sentence pairs not very similar when the embeddings were trained on a small corpus. For example, it was easy to determine that “it be appropriate to retain the patient at the present level of care since the patient be make progress but have not yet achieve the goal articulate in the individualize treatment plan” and “the patient demonstrates the ability to fire the ta g and fhl of the operative extremity” are not semantically similar (i.e., similarity of 0) when we applied the system using one-hot representation as there was no *N*-gram overlapped by the two sentences, but a little semantically similar (e.g., similarity of 2.02 when using CNN) when we applied the system using CNN or Bi-LSTM. The improvement because of concatenation or fusion of distributed representation and one-hot representation mainly came from sentence pairs of high similarity. As an illustration, we compared mean square error (MSE) on fractional similarity intervals as shown in Fig. 3.

For further improvement, there are three possible directions as follows: 1) fuse multiple distributed representations with one-hot representation as different distributed representations may be complementary; 2) increase more data for word embedding training and model training; 3) introduce domain knowledge into our framework. All of them will be investigated in the future.

## Conclusion

In this paper, we proposed a novel framework to fuse distributed representation and one-hot representation using a gated network for clinical STS. Experiments on a benchmark dataset showed that the two types of representations were complementary and gated network was a good way for representation fusion.

## Data Availability

Our annotated corpus was supplied by BioCreative/OHNLP oraganization on clinical semantic textual similarity shared task.

## Abbreviations

BERT: Bidirectional encoder representations from transformers
Bi-LSTM: Bi-directional long short term memory networks
CNN: Convolutional neural network
EHR: Electronic health records
IDF: Inverse document frequency
IR: Information retrieval
NLP: Natural language processing
QA: Question answer
STS: Semantic textual similarity
